# Supplementary material for: Wide Real-Life Data Support Reduced Sensitivity of Antigen Tests for Omicron SARS-CoV-2 Infections
Source: Viruses. 2024 Apr 23;16(5):657. doi: 10.3390/v16050657 (PMC11125898; doi:10.3390/v16050657)
Supplement: Supplementary file 1 [file viruses-16-00657-s001.zip › viruses-2940536-supplementary.pdf]

Supplementary materials

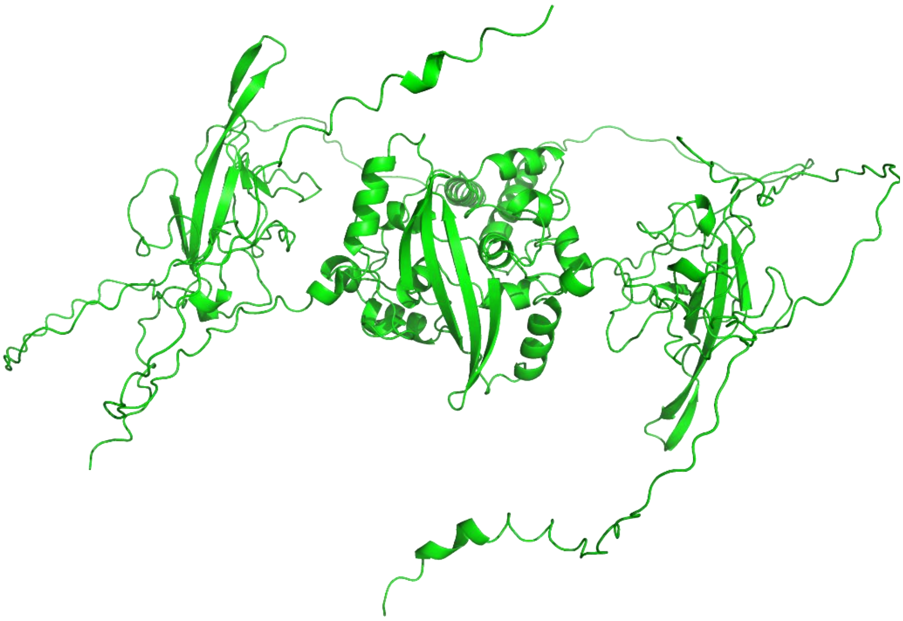

Supplementary Figure S1. Model of the full-length dimer N protein using AlphaFold2.

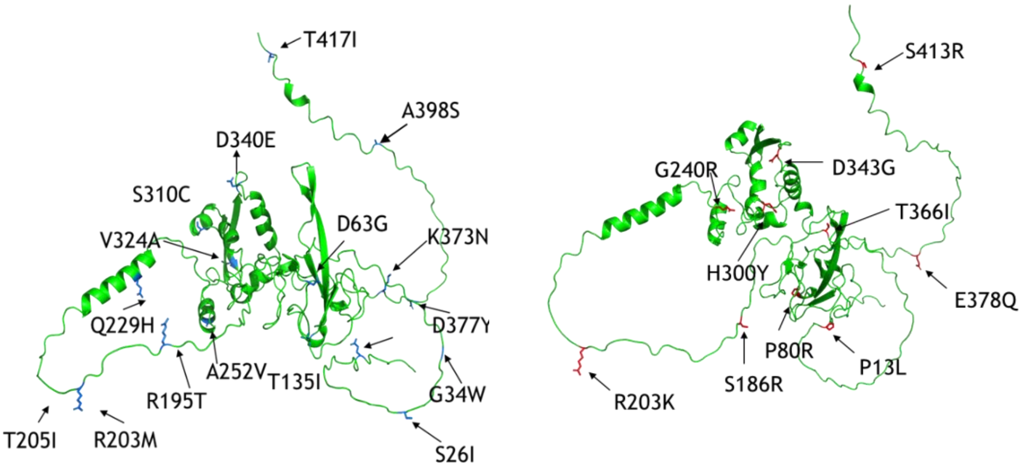

Supplementary Figure S2. Mutations mapped in the N monomer protein structure predicted by AlphaFold. The mutations are color-coded with blue representing those exclusive to the Delta variant, red representing those exclusive to the Omicron variant.

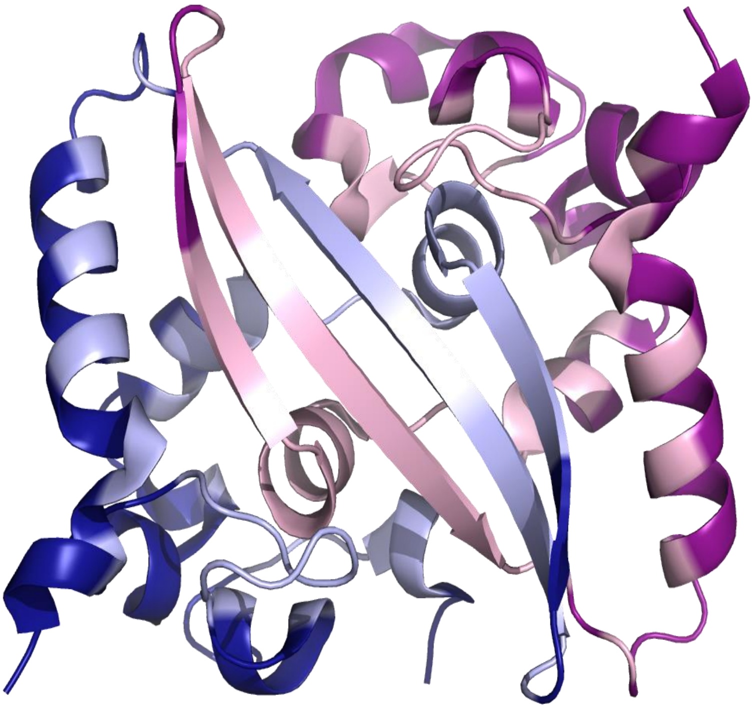

Supplementary Figure S3. CTD domain dimer of the N protein structure. Light pink: residues belonging to the dark blue chain (A). Light purple: residues belonging to the dark purple chain (B). CTD domain PDB ID: 6wzo.

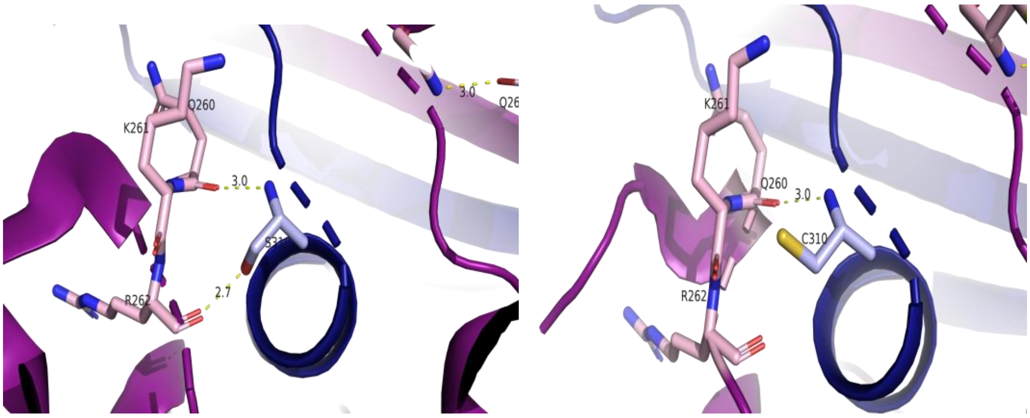

Supplementary Figure S4. Zoom in showing the S310 mutated into Cysteine (S310C) and re-evaluation of the interactions after the substitution. On the left side, interactions occur involving the residue positioned at Ser310 with Gln 260, Lys 261, and Arg 262. On the right, there is a representation of amino acid substitution. We observed only minor difference of one less bond between Arginine 262 and Cysteine.

Mutant	PDB id	ΔΔG (kcal/mol) DynaMut2	ΔΔG (kcal/mol) DynaMut	ΔΔSvibENCoM (kcal.mol-1.K-1)
P80R	6vyo	0,41 kcal/mol	1,460 kcal/mol	-0.522 kcal.mol^{-1}.K^{-1}
H300Y	6wzo	1,16 kcal/mol	0.895 kcal/mol	-0,131 kcal.mol^{-1}.K^{-1}
S310C	6wzo	-0,43 kcal/mol	-0.217 kcal/mol	0.113 kcal.mol^{-1}.K^{-1}
D343G	6wzo	-0,28 kcal/mol	-0.958 kcal/mol	0.130 kcal.mol^{-1}.K^{-1}

Table. S1. ΔΔG and ΔΔSvibENCoM calculations using DynaMut and DynaMut2 webservers. The positive ΔΔG indicates increased stability, while negative ΔΔG indicates decreased stability. A negative ΔΔSVibENCoM implies an increase in protein rigidification, while a positive ΔΔSVibENCoM implies an increase in the flexibility of protein structure.

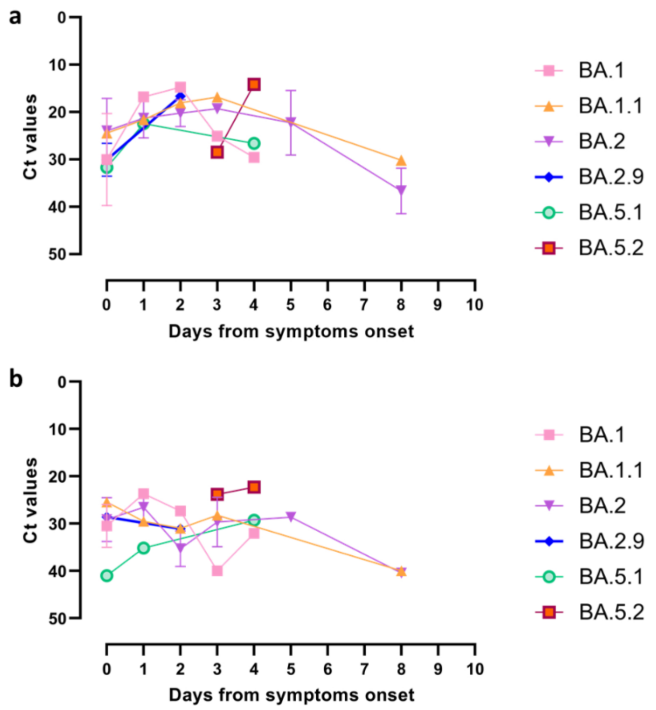

Supplementary Figure S5: Mean Ct values and Standard Deviation for each SARS-CoV2 lineage detected in the nose (A) and in the mouth (B) based on days after symptoms onset.
